# Impact of a novel pharmacist-delivered behavioral intervention for patients with poorly-controlled diabetes: The ENhancing outcomes through Goal Assessment and Generating Engagement in Diabetes Mellitus (ENGAGE-DM) pragmatic randomized trial

**DOI:** 10.1371/journal.pone.0214754

**Published:** 2019-04-02

**Authors:** Julie C. Lauffenburger, Roya Ghazinouri, Saira Jan, Sagar Makanji, Christina A. Ferro, Jennifer Lewey, Eric Wittbrodt, Jessica Lee, Nancy Haff, Constance P. Fontanet, Niteesh K. Choudhry

**Affiliations:** 1 Center for Healthcare Delivery Sciences (C4HDS), Department of Medicine, Brigham and Women’s Hospital and Harvard Medical School, Boston, Massachusetts, United States of America; 2 Division of Pharmacoepidemiology and Pharmacoeconomics, Department of Medicine, Brigham and Women’s Hospital and Harvard Medical School, Boston, Massachusetts, United States of America; 3 Horizon Blue Cross Blue Shield of New Jersey, Newark, New Jersey, United States of America; 4 Rutgers State University of New Jersey, New Brunswick, New Jersey, United States of America; 5 Magellan Rx Management, Newport, Rhode Island, United States of America; 6 Division of Cardiovascular Medicine, Hospital of the University of Pennsylvania, Philadelphia, Pennsylvania, United States of America; 7 AstraZeneca, US-Medical Affairs, Wilmington, Delaware, United States of America; Third Military Medical University Daping Hospital and Research Institute of Surgery, CHINA

## Abstract

**Background:**

Many factors contribute to suboptimal diabetes control including insufficiently-intensive treatment and non-adherence to medication and lifestyle. Determining which of these is most relevant for individual patients is challenging. Patient engagement techniques may help identify contributors to suboptimal adherence and address barriers (using motivational interviewing) and help facilitate choices among treatment augmentation options (using shared decision-making). These methods have not been used in combination to improve diabetes outcomes.

**Objective:**

To evaluate the impact of a telephone-based patient-centered intervention on glycosylated hemoglobin (HbA1c) control for individuals with poorly-controlled diabetes.

**Design:**

Two-arm pragmatic randomized control trial within an explanatory sequential mixed-methods design.

**Subjects:**

1,400 participants 18–64 years old with poorly-controlled type 2 diabetes.

**Intervention:**

The intervention was delivered over the telephone by a clinical pharmacist and consisted of a 2-step process that integrated brief negotiated interviewing and shared decision-making to identify patient goals and options for enhancing diabetes management.

**Main measures:**

The primary outcome was change in HbA1c. Secondary outcomes were medication adherence measures. Outcomes were evaluated using intention-to-treat principles; multiple imputation was used for missing values in the 12-month follow-up. We used information from pharmacist notes to elicit factors to potentially explain the intervention’s effectiveness.

**Key results:**

Participants had a mean age of 54.7 years (SD:8.3) and baseline HbA1c of 9.4 (SD:1.6). Change in HbA1c from baseline was -0.79 (SD:2.01) in the control arm and -0.75 (SD:1.76) in the intervention arm (difference:+0.04, 95%CI: -0.22, 0.30). There were no significant differences in adherence. In as-treated analyses, the intervention significantly improved diabetes control (-0.48, 95%CI: -0.91, -0.05). Qualitative findings provided several potential explanations for the findings, including insufficiently addressing patient barriers.

**Conclusions:**

A novel telephone-based patient-centered intervention did not improve HbA1c among individuals with poorly-controlled diabetes, though as-treated analyses suggest that the intervention was effective for those who received it.

**Trial registration:**

ClinicalTrials.gov NCT02910089

## Introduction

Even though it is well established that good glycemic control reduces mortality and long-term complications, more than 40% of patients with type 2 diabetes (T2D) do not achieve their glycosylated hemoglobin (HbA1c) goals.[1–3] Suboptimal disease control could result from patient non-adherence to prescribed medication or lifestyle modifications, the failure of providers to appropriately intensify therapy when indicated, or a combination of these factors.[4, 5] Unfortunately, providers often cannot easily identify which of these factors is most relevant for individual patients.[6, 7]

Several patient interviewing techniques have been developed to elicit barriers that patients perceive as being important for their care.[6] Brief negotiated interviewing is a form of motivational interviewing that involves short counseling sessions of feedback, advice, and motivational enhancement techniques.[8, 9] It has shown promising results in improving adherence and decreasing unhealthy behaviors such as alcohol abuse.[9–12] In the case of T2D, brief negotiated interviewing may help identify whether treatment intensification or adherence improvement would be a more effective approach to improve disease control and help address barriers to adherence when they exist. Conversely, if treatment intensification is necessary, other techniques such as shared decision-making, a collaborative process between patients and providers, may help elicit patient preferences for which therapies are most congruent with patients’ goals.[8, 13, 14] While brief negotiated interviewing and shared decision-making complement each other, no studies have combined these approaches. Moreover, few studies have delivered either of these techniques in a manner that could be scaled to large populations, such as over the telephone, even though health insurers routinely use this type of outreach to contact their beneficiaries.[15–17]

Therefore, we launched the ENhancing outcomes through Goal Assessment and Generating Engagement in Diabetes Mellitus (ENGAGE-DM), a pragmatic, prospective, open-label, intention-to-treat randomized controlled trial.[8] This trial sought to evaluate whether using a novel telephone-based patient-centered intervention improved HbA1c control for patients with poorly-controlled diabetes. We used an explanatory sequential mixed-methods framework[18], in which we first evaluated the impact of the intervention and then elicited factors that could potentially explain its effectiveness.[18]

## Methods

### Quantitative methods

#### Study design

Details of the study design and protocol have been published previously.[8] The trial was approved by the Institutional Review Board (IRB) of Brigham and Women’s Hospital and the privacy board of Horizon Blue Cross Blue Shield of New Jersey (“Horizon”) and is registered with clinicaltrials.gov (NCT02910089). A waiver of informed consent and authorization to use the study data was granted by the IRB and Horizon privacy board because of the minimal risk nature of the study and because the research could not have feasibly been done without the waiver or access to potentially-identifiable information. The trial protocol was designed, written, and executed by the investigators (S1 Protocol). We designed the trial to be pragmatic using PRECIS-2 trial guidance and reported using CONSORT trial guidelines (S1 Checklist).[19] The authors analyzed the trial data using an independent copy of the study database and vouch for analytic accuracy and fidelity to the study protocol. Study enrollment began in October 2016 and was completed in December 2016. Follow-up of all trial participants ended in December 2017. Final data were received by June 2018.

#### Study setting and population

The trial was conducted at Horizon Blue Cross Blue Shield of New Jersey (Horizon), the largest health insurer in New Jersey. We included patients ≥18 years of age who filled 1 or more oral hypoglycemic agents within the 12 months prior to randomization and who had evidence of poor diabetes control (defined as HbA1c ≥8%).[2, 4] Horizon regularly receives this type of laboratory information from over 200 patient-centered medical homes and population health programs as part of routine quality improvement monitoring. Patients were excluded if they were insured by Medicaid or Medicare, had <3 months of continuous enrollment, had recently filled insulin, or had no telephone contact information (which would prevent delivery of the intervention). An HbA1c minimum threshold of 8% was chosen based being the minimum control threshold for major quality measures for health plans.[3, 20]

#### Randomization and study arms

Subjects were randomized by Horizon in a 1:1 ratio to the intervention or usual care group using a random number generator. Randomization occurred at the patient level.

The primary component of the multi-faceted intervention was an individually-tailored telephone consultation conducted by a clinical pharmacist. Patients randomized to the intervention group were sent an invitation letter informing them about the study, including a simple pillbox and a shared decision-making tool to prime the patients for the telephone consultations. This tool was postcard-sized and was developed using principles of decision aid design, querying patients about how they manage their diabetes.[13] Within two weeks of the mailing, patients were contacted by telephone to participate in the consultation or schedule it for a later date. Patients were asked to provide verbal consent for the clinical pharmacist consultation.

The consultation was conducted using a semi-structured call guide developed by the study team with direct input from patients. The interventions were delivered by trained pharmacists from Magellan Rx Management, a pharmacy benefit management company that specializes in pharmacist-delivered telephonic disease management services. Prior to study launch, the pharmacists underwent a training program that included script development and role-playing exercises.

The overall structure of the behaviorally-tailored telephone intervention was based on the principles of brief negotiated interviewing.[8, 9, 21, 22] The telephone consultation began with a review of study medications and then proceeded into a conversation eliciting the reasons for poor disease control, using open-ended questions to identify patient preferences. Based on this, patients identified one of 3 strategies to improve their diabetes control: 1) treatment intensification, 2) adherence improvement, or 3) lifestyle improvement. Patients could also identify being ‘not ready’ to select a strategy at present as a fourth option.

The subsequent interventions were tailored based on the strategy that each patient chose. For patients who chose to focus on improving medication adherence or lifestyle modifications, a second, separate brief negotiated interviewing process was used to help patients identify their adherence barriers and to propose solutions tailored to their specific barriers. These solutions included pillboxes, simple alarm reminders, mail order use, dietitian support, counseling, or other exercise recommendations. For patients who chose treatment intensification, shared decision-making was used to identify treatment options they would prefer.[13, 14] For patients who were not ready to select a strategy, the pharmacists repeated the consultation at a follow-up call. For all patients who selected a strategy, the proposed plan was communicated by the pharmacist to the patient’s provider via fax. Follow-up “booster” phone calls by the pharmacists were used up to 3 more times, which continued to engage the patient in discussions about their barriers to disease control. The pharmacists were also provided patients’ updated HbA1c laboratory data from Horizon throughout the 12-month follow-up period in order to better tailor the discussions.

Patients randomized to the usual care arm were not contacted in any way.

Study subjects and pharmacists interacting with patients were not blinded to group assignment. Study investigators and data analysts remained blinded until all follow-up data were obtained, and the analyses were finalized.

#### Study outcomes

The trial’s primary outcome was the change in HbA1c from baseline (i.e., the closest value before randomization) to 12 months after randomization. In keeping with the pragmatic nature of the trial[19], HbA1c was assessed using laboratory values collected as part of routine care, rather than at study-specific visits.[23] The HbA1c result recorded closest to the end of the 12-month follow-up was used. As expected with the use of routinely-collected data, clinical HbA1c data was missing for some (28.6%) patients, including 28.7% in the usual care arm and 28.5% in the intervention arm. Accordingly, we used multiple imputation to handle the missing data.[24] In specific, we used 20 imputations with Proc MI in SAS version 9.4 (Cary, NC) to fill in any estimated values using fully conditional specification. Analyses were then conducted on each imputed dataset using generalized estimating equations with an identity link and normally-distributed errors. The results from each analyzed dataset were combined using Rubin’s rules.[24] This approach achieved in-range values and a 98% relative efficiency.

The prespecified secondary outcomes included the proportion of patients achieving HbA1c <8%[3] and medication adherence in the 12-month follow-up period. Adherence was measured by linking all observed fills based on dispensing date and days’ supply. Different drugs in the same chemically-related therapeutic class (e.g., sulfonylureas) were considered to be interchangeable.[25] From these supply diaries, we calculated the proportion of days that patients had ≥1 oral glucose-lowering medication available, or the proportion of days covered (PDC), by dividing the number of days with medication available by the number of days during follow-up.[26] This adherence metric is used by Medicare Star ratings and health plans for quality improvement.[27] Patients were censored when they lost continuous eligibility. Medication adherence was measured as both a continuous measure and as the proportion of patients achieving optimal adherence (≥80%).[8, 25, 26] There was no missing data for the adherence outcomes.

#### Statistical analysis

We randomized 1,400 patients in a 1:1 ratio to achieve more than 80% power to detect an average HbA1c change of 0.5% between the intervention and usual care arms (alpha = 0.05, HbA1c standard deviation of 1.9, two-sided significance).[8] We powered the study assuming that approximately 30–35% of subjects in the intervention arm would agree to participate. Prior studies of telephonic pharmacist interventions have indicated a 30–55% acceptance rate by patients.[15, 28] Using Proc Power (two-sample means) in SAS version 9.3 (Cary, NC), we estimated that we would need 228 subjects per arm, prior to factoring in an estimated 30–35% acceptance rate in the intervention group (i.e., the midpoint of this range, 33%, would require 691 patients/group). Therefore, approximately 700 study subjects per arm were therefore estimated to be necessary after considering the estimated acceptance rate.

For the analyses, we used intention-to-treat principles for all randomized patients, i.e., regardless of whether patients actually received the intervention. We calculated the means and frequencies of pre-randomization variables separately by arm and compared them using absolute standardized differences.

We evaluated change in mean HbA1c using generalized estimating equations with an identity link and normally-distributed errors. Analyses of medication adherence were compared using an identify link function and normally distributed errors.

We also conducted additional sensitivity and secondary analyses, including adjustments for imbalanced baseline covariates with a standardized difference >0.1. For HbA1c, we conducted a “complete case” analysis. Subgroup analyses were performed according to age, sex, and baseline HbA1c control. For adherence, we measured PDC beginning with the first fill after randomization, censored patients upon initiation of insulin, and measured average adherence across all eligible diabetes medications.

We also conducted an “as-treated” analysis, following recommended approaches for pragmatic trials, in which we used propensity score matching to identify patients in the usual care arm who had similar sociodemographic and baseline characteristics as those reached in the intervention arm.[29–31] In this approach, we first calculated a propensity score by estimating the probability of receiving the intervention using logistic regression including all baseline covariates, which were selected based on literature and expert opinion. After estimating the propensity score, few patients had propensity scores that did not overlap with patients in the other group. We excluded these patients prior to matching to trim the distribution. We then used a greedy matching algorithm (5:1 digit matching) to 1:1 match patients to provide estimates for the average effect of the intervention in treated individuals.[30, 32] Absolute standardized differences and post-matching C-statistics were compared for the characteristics.

In exploratory analyses, among patients receiving the intervention, we also evaluated differences in effectiveness of the intervention by which of the four decisions patients made to control their diabetes during the consultation, using ANOVA. We hypothesized that the effectiveness could differ among these groups.

### Qualitative methods

#### Design and sampling

We reviewed full-text copies of the detailed notes recorded by clinical pharmacists during their initial consultation with patients. We received detailed notes for 96% of all patients who received the initial consultation. Each of the notes, in paragraph form, described which diabetes medications patients were taking, how they are managing their diabetes, their self-reported barriers to optimal glycemic control, their rationale for the strategy they chose (e.g., adherence improvement), their readiness to change, and the shared plan developed with the clinical pharmacist. Many notes included direct quotations from patients. All patient identifiers were removed prior to sharing with the study investigators.

#### Analysis

We used a grounded theory approach to evaluate the clinical pharmacist notes. The objective was to elicit factors that could be explain patient’s reasons for their chosen strategy and identify themes associated with the effectiveness of the intervention. In this approach, the notes were coded and analyzed using constant comparative analysis to generate themes.[33] Verbatim quotations from patients and pharmacists were identified that supported or further elaborated on an identified theme. ATLAS.ti qualitative data analysis software was used to help identify the themes (Berlin, Germany). Because these types of telephonic quality improvement interventions are used regularly by healthcare practices and insurers, we combined the qualitative and quantitative findings to provide hypotheses for how to improve the intervention.

## Results

### Quantitative findings

**Fig 1:**
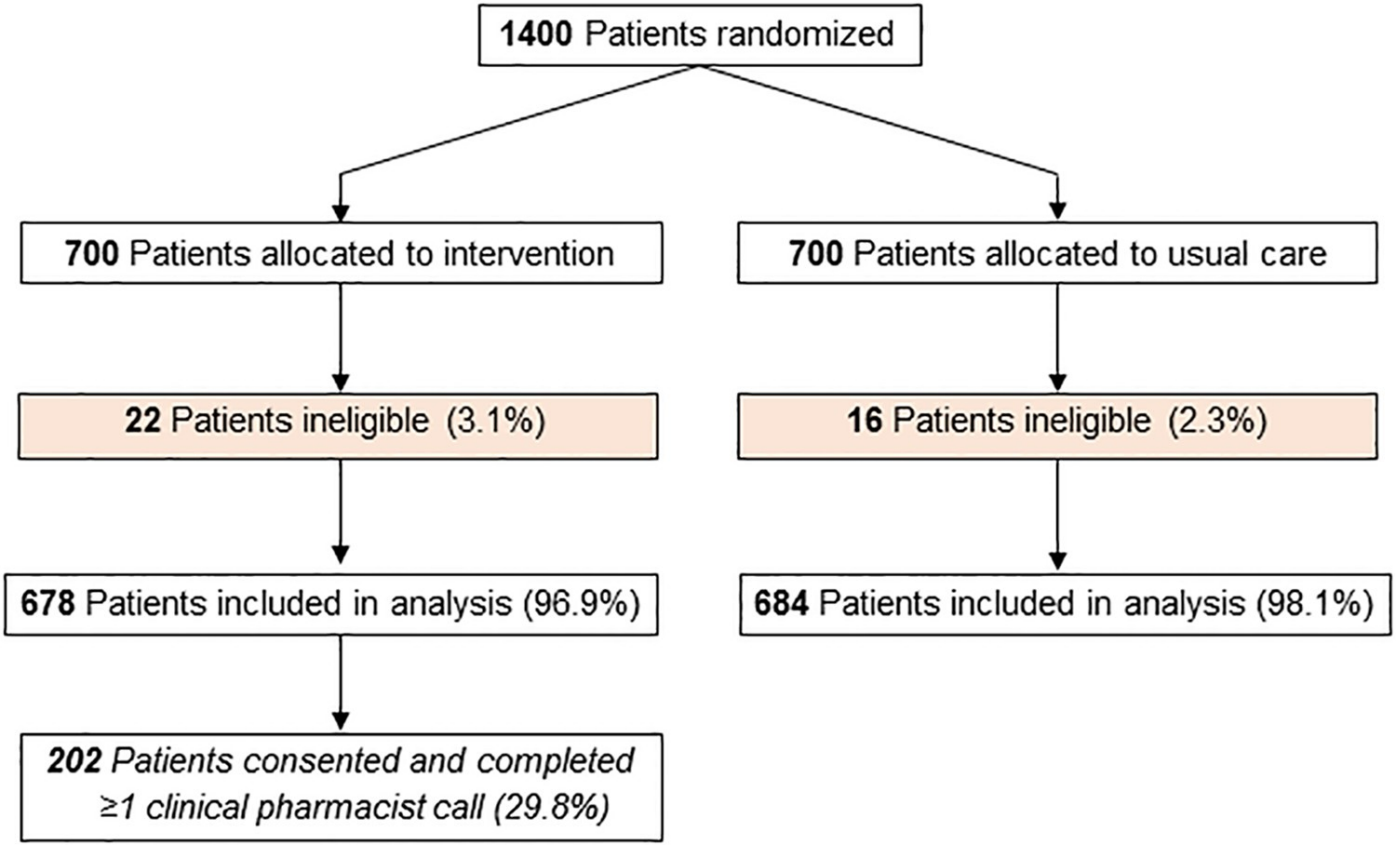
Flow diagram of patients through the trial.

Of the 1,400 randomized patients, participants had a mean age of 54.7 years (SD: 8.3), baseline HbA1c of 9.4 (SD: 1.6), and 37.2% were female. Baseline characteristics are shown in Table 1. Intervention patients were slightly less likely to be female and slightly more likely to have had a prior stroke/transient ischemic attack but were otherwise similar to usual care patients, including baseline HbA1c and medication adherence. In total, 38 (2.8%) were not included in the analysis because they lost insurance eligibility between the time of data pull and randomization, which was the beginning of follow-up (Fig 1).

**Table 1:** Patient characteristics by study arm.

Baseline characteristics	Usual Care (n = 700)	Intervention (n = 700)	Absolute standardized differences
**Demographic**			
Age, mean (SD)	54.6 (8.4)	54.9 (8.1)	0.01
Female gender, %	39.8	34.6	0.13
**Diabetes values**			
HbA1c, mean (SD)	9.4 (1.6)	9.3 (1.6)	0.02
**Oral hypoglycemic use and adherence**			
No. oral hypoglycemics, mean (SD)	2.1 (1.0)	2.1 (1.0)	0.05
Concomitant non-insulin injectable, %	11.7	11.1	0.03
Adherence, mean (SD)	79.8 (22.1)	80.5 (21.3)	0.00
Copayment, mean (SD)	35.1 (63.9)	36.5 (70.6)	0.02
Type of medication			
Generic only, %	47.1	47.8	0.02
Mixture, %	41.5	40.0	0.04
Brand only, %	11.4	12.2	0.03
**Diabetes characteristics, %**			
Hypoglycemia	0.3	0.4	0.03
Retinopathy	3.8	3.4	0.03
Neuropathy	55.9	54.2	0.04
**Other clinical characteristics, %**			
Coronary artery disease	11.0	12.3	0.06
Hypertension	69.4	71.6	0.05
Hyperlipidemia	65.6	67.6	0.06
Congestive heart failure	0.9	0.9	0.00
Stroke/transient ischemic attack	2.8	4.0	0.10
Obesity	26.3	27.1	0.02
Asthma/COPD	8.9	9.7	0.04
Liver disease	8.4	8.3	0.00
Chronic kidney disease	51.0	50.0	0.02
Depression	5.6	4.7	0.05
Acute stress	1.8	1.6	0.02
Combined comorbidity score, mean (SD)	0.7 (1.4)	0.6 (1.4)	0.05
**Resource utilization**			
ER visits, mean (SD)	0.2 (0.8)	0.2 (0.6)	0.03
No. of days hospitalized, mean (SD)	0.3 (2.0)	0.5 (3.3)	0.02
Office visits, mean (SD)	7.2 (6.2)	7.0 (5.2)	0.01

Abbreviations: SD, Standard Deviation; COPD, Chronic obstructive pulmonary disease; HbA1c, glycosylated hemoglobin A1c

In total, 202 (29.8%) intervention patients completed an initial telephone consultation with a clinical pharmacist; each telephone consultation lasted an average of 30 minutes. The mean number of days between the baseline HbA1c and randomization was 74.0 days (SD: 42.7) and between randomization and the first consultation was 83.1 days (SD: 37.8). Among those completing the initial consultation, 106 (52.5%) patients received a second call and 52 (25.7%) received ≥3 calls. During this consultation, 78 (38.6%) reported that they were adherent to the prescribed medications and 40 (19.8%) reported that their diabetes therapy regimen had recently changed. Within the intervention arm, the pre-randomization characteristics of those who received the clinical pharmacist intervention compared with those are shown in S1 Table.

The strategy patients felt would be most beneficial for optimizing their glycemic control was lifestyle improvement for 92 (45.5%) patients, medication adherence for 32 (15.8%) patients and treatment intensification for 28 (13.9%) patients. During the initial consultation, 50 (24.8%) patients were not ready to make a change to their diabetes management or regimen. Of the patients choosing to focus on medication adherence improvement, the pharmacists recommended changing to longer dispensations of medications for 14 patients and use of mail order in 3 patients.

The mean change in HbA1c from baseline to follow-up was -0.79 [SD: 2.01]) for control and -0.75 [SD: 1.96] for intervention subjects (between group difference: +0.04, 95% Confidence Interval [CI]: -0.22, 0.30) (Table 2). A complete case analysis found similar results (S2 Table). The results remained unchanged after adjusting for imbalances in baseline characteristics. In the propensity-score matched ‘as-treated’ analyses comparing 196 similar patients in each arm, the mean pre- to post-change in HbA1c was -0.48 (1.73) for control and -0.96 (SD: 1.69) for intervention (difference: -0.48, 95%CI: -0.91, -0.05) (S3 and S4 Tables).

**Table 2:** Primary outcome by study arm.

Outcome	Usual Care (n = 684)	Intervention (n = 678)	Unadjusted	Adjusted*
**Change in glycemic control**^§^			**Absolute difference (95% CI)**	
Mean change in HbA1c, mean (SD)	-0.79 (2.01)	-0.75 (1.96)	+0.04 (-0.22, 0.30)	+0.06 (-0.20, 0.32)

*Adjusted for sex and prior stroke/transient ischemic attack

^§^Using multiple imputation (28.7% and 28.5% missing in usual care and intervention, respectively)

Abbreviations: HbA1c, glycosylated hemoglobin A1c; SD, Standard Deviation; CI, Confidence interval; PDC, proportion of days covered

Subgroup analyses are shown in S5 Table. Although there were also no significant differences between groups, men tended to respond better to the intervention than women. Among those receiving the intervention, we observed that patients choosing adherence or lifestyle improvement tended to have larger changes in disease control than those that chose treatment intensification or were not ready to make a decision (S6 Table), though the differences were also not significant.

Analyses of secondary outcomes are presented in Table 3. Mean (SD) adherence during follow-up in patients allocated to usual care and intervention was 66.2% (SD: 32.7) and 67.5% (SD: 31.6), respectively, resulting in no significant between-group differences (1.25%, 95%CI: -2.2%, 4.7%). As-treated analyses observed between-group differences of +4.4% (-1.4%, 10.3%) in mean follow-up adherence (S4 Table). Sensitivity analyses of the adherence outcome definition are shown in S7 Table and yielded results very similar to the primary results.

**Table 3:** Secondary outcomes by study arm.

Outcome	Usual Care (n = 684)	Intervention (n = 678)	Unadjusted	Adjusted*
**Proportion achieving optimal HbA1c**			**Odds ratio (95% CI)**	
HbA1c<8.0% in follow-up^§^, %	38.0%	34.8%	0.92 (0.72, 1.18)	0.91 (0.71, 1.17)
**Medication adherence: PDC**			**Absolute difference (95% CI)**	
Adherence to ≥1 oral glucose lowering medication, mean (SD)	81.9 (31.0)	81.9 (30.1)	-0.03 (-3.27, +3.20)	-0.16 (-3.41, +3.08)
**Proportion achieving optimal adherence (PDC ≥80%)**			**Odds ratio (95% CI)**	
Adherent to ≥1 medication, %	73.7%	72.1%	0.92 (0.73, 1.17)	0.92 (0.72, 1.17)

*Adjusted for sex and prior stroke/transient ischemic attack

^§^Using multiple imputation (28.7% and 28.5% missing in usual care and intervention, respectively)

Abbreviations: HbA1c, glycosylated hemoglobin A1c; SD, Standard Deviation; CI, Confidence interval; PDC, proportion of days covered

### Qualitative findings

In total, 193 patients (95.5%) had detailed clinical pharmacist notes. Qualitative analysis of the clinical notes produced several themes that could help explain the effectiveness of the intervention. These themes are summarized in Table 4 with representative quotations for each of these six themes.

**Table 4:** Emergent themes about the effectiveness of the intervention.

Theme	Representative quotations	Potential mechanism influencing effectiveness of the intervention
1) Patients often attributed their poor disease control to their inability to consistently manage their diet.	*- “He stated diet was his biggest issue as he likes to eat*, *and he has seen a nutritionist twice already*.*”* *- “Dieting is difficult for him… He has to go find food most of the time and finds that he is still hungry after eating*.*”*	The intervention was designed to deliver strategies ranging from adherence support to treatment intensification but may not have provided sufficiently intensive dietary support for some patients.
2) Patients attributed their poor disease control to difficulty exercising.	*- “It’s harder in cold months because she has a bad knee*, *but she is mobile and on her feet at work*.*”* *- “He tries to walk every day and he is also on his feet for work*. *He has had some back issues*, *so it is hard to do other exercise*.*”*	The clinical pharmacists could provide any type of necessary counseling but may not have provided sufficiently intensive exercise support for some patients.
3) Patients had recently changed their treatments or lifestyle and were waiting on their next appointment or lab test.	*- He “started the exercise and diet changes after this lab result”*. *- “he wants to see what ‘his numbers’ are at that appointment before he makes any major changes to therapy*.*”* *- This patient “lost around 40lbs in the last 6 months”*.	The delay that sometimes occurred between the receipt of the HbA1c lab test and the delivery of the intervention may have reduced the accuracy of these values and, as a result, the effectiveness of the intervention.
4) Pharmacists documented when they had reached patients who were at work to explain the nature of the conversation.	- The pharmacists frequently noted when the member was at work. - Several pharmacists noted that the conversation was *“rushed”*.	The intervention was primarily delivered during the work day; while patients could schedule it for a convenient day, the consultation may not have been optimally delivered as they may not have been fully engaged in the conversation.
5) Patients sometimes chose treatment intensification even though they appeared to really need to focus on lifestyle adherence.	- *“She tries to manage her diet… and also tries to get some exercise but that is hard right now”* so *“she chose treatment intensification”*. *- “She tries to watch her carb intake and doesn’t have a lot of time for exercise or to follow a specific treatment”* but she *“was interested in treatment intensification”*.	Patients may not have chosen the strategy that clinically was the most necessary for them, particularly if other behavioral changes could have potentially greater impact.
6) Patients were hesitant to add on a new therapy, especially insulin, and were more receptive to escalations in dose.	- “*She mentioned that Dr*. *[x] wants to start her on insulin*, *but she is hesitant and wants to improve her diabetes with her diet*.*”* - She *“was ready to increase the dose of his medication but would not like to add any*”.	Providers may not have agreed with the chosen strategy. If insulin is the most optimal intensification strategy, providers may need to deliver additional intervention to address patient concerns.

#### Theme 1: Patients attributed their poor disease control primarily to their inability to consistently manage their diet

Most patients cited dietary choices as the hardest part about managing their diabetes. Patients reported prior meetings with dietary educators and physicians but expressed difficulty consistently adhering to a guideline-concordant diet.

#### Theme 2: Patients attributed their poor disease control to difficulty exercising

Patients reported difficulty getting enough exercise given their work and life schedules. Moreover, the weather (i.e., winter) was very commonly referred to in conversations with the clinical pharmacists; many patients noted that they were deferring until January or the spring to get active again so that they could walk outside.

#### Theme 3: Some patients had recently changed their treatments or lifestyle and were waiting for their next appointment or lab test

Numerous patients were not ready to choose a strategy with a clinical pharmacist, citing that they had a near-term appointment with their doctor or were waiting until their next laboratory measure. Some patients had made significant changes since their last visit.

#### Theme 4: Pharmacists documented when they had reached patients who were at work to explain the nature of the conversation

Several patients were taking a break from work while the intervention was being delivered. On some of these occasions, pharmacists documented when they felt like the conversation was rushed.

#### Theme 5: Patients sometimes chose treatment intensification even though they really needed to focus on lifestyle adherence

On a few occasions, pharmacists described that patients reported difficulty with lifestyle management but that the patients were interested in pursuing treatment intensification with their providers. Some of these patients reported regular adherence to medications but trouble with their diet or exercise.

#### Theme 6: Patients were hesitant to add on a new therapy, especially insulin and were sometimes more receptive to escalations in dose

In several encounters, the pharmacists commented on how patients wanted to avoid insulin but were more receptive to combination oral medications or increasing the dose of medications to avoid adding any new medications.

## Discussion

In this pragmatic trial of patients with poorly-controlled diabetes, we found that the most common reason that patients identified for their lack of disease control was poor diet or exercise and that despite having poorly-controlled disease, 25% of patients were not ready to change the way they currently manage their diabetes or their regimen. In this context, a patient-centered telephonic intervention that combined motivational interviewing and shared decision-making principles did not improve HbA1c when evaluated using intention-to-treat principles. In contrast, in as-treated analyses, the intervention resulted in a significant reduction in glycosylated hemoglobin.

This trial was motivated by evidence from prior studies demonstrating that pharmacist-delivered telephone interventions improve medication adherence and other patient outcomes.[6, 17, 28] Health insurers and practices routinely use this type of outreach for their beneficiaries for medication therapy management and adherence interventions in clinical practice.[34, 35] We leveraged this approach and combined two patient engagement techniques, brief negotiated interviewing and shared decision-making, which address the complementary goals of adherence enhancement and treatment intensification that are relevant to patients with poorly-controlled diabetes.[8, 9, 13, 14] Despite this theoretical rationale, the intervention was ineffective in the intention-to-treat analysis. There are several explanations for this result that stem from the qualitative analyses we conducted.

First, the intervention may not have adequately addressed their perceived or real primary barriers to disease control. Patients attributed their poor disease control most commonly to poor diet or exercise habits, even though the intervention was largely designed to address therapeutic issues, such as adherence or treatment intensification. While the pharmacists educated patients on diet and exercise strategies it is probable that the specificity of this guidance was insufficient to result in better disease control. It is also possible that the intervention may not have worked as well for patients with comorbid conditions like kidney or liver disease or asthma/COPD, due to physiologic differences in how these conditions affect glycemic control. Moreover, patients ultimately drove the selection of strategy, with guidance from the pharmacist, and this choice may not have addressed the factor that was most necessary to address. These explanations are supported by the observations that numerous patients reported diet or exercise as their primary barriers and that those choosing treatment intensification tended to have smaller HbA1c reductions. Alternatively, while the outreach was being done by trained clinical pharmacists who were skilled at engaging patients by telephone, these individuals were not a regular part of patients’ clinical care teams.[28] These results should not be interpreted as evidence that pharmacists, in general, are ineffective at helping patients address non-adherence.

Second, a quarter of patients were not ready to make a change to their therapeutic strategy despite having poorly-controlled diabetes. While several participants indicated that they were waiting for updated HbA1c values or their upcoming doctor’s appointment to guide decision making or that they had already made a significant change in their lifestyle (e.g., losing weight), still others were not sure or not ready to choose a strategy for less clear reasons. It could be that patients were resistant to change, for example, being in early stages of behavior change. Patients were also often hesitant to add on a new therapy and may not have been ready to choose treatment intensification.

Third, the intervention relied on lab values that came from healthcare practices. For many patients, there was a substantial gap between the HbA1c value used for enrollment and the pharmacist consultation. In this interval, patients may have made changes to their treatment regimens or improved adherence to medications thereby making the pharmacist consultation less relevant for these individuals. These delays could have been due to the lag in provider organizations providing laboratory values to Horizon, the time required to process the data for patient outreach, and the nature of how often HbA1cs are measured (i.e., approximately every 3 months). The qualitative analysis corroborated this explanation, in that some patients addressed self-acknowledged barriers to disease control before the intervention was administered while others had recently changed their treatments and were waiting for their next doctor’s appointment or lab test to guide next steps.

Fourth, we analyzed the effectiveness of the targeted interventions among all patients randomized to the study arms using intention-to-treat principles, even though only 30% of patients accepted the initial pharmacist consultation. The contact rate we achieved, while somewhat low, was similar to other telephonic quality improvement interventions delivered by pharmacists[15, 28] and mirrors how this type of intervention is delivered in real-world settings. In contrast, if we had randomized only those patients who had agreed to participate in the intervention, our findings would not have been fully representative to the population to whom it would ultimately be applied (i.e., patients identified for intervention through the review of insurance data).[6, 36] Moreover, no specific intervention was provided to the usual care arm. As such, while we accounted for the fact that a minority of randomized subjects would agree to the consultation in our power calculations, this may, nevertheless, have undermined our ability to see a true effect.

Accordingly, while the intention-to-treat effect evaluates the effectiveness of the intervention on average in the population, clinicians, healthcare organizations, and patients are often interested in knowing whether an intervention works for those who actually receive it.[37] Our “as treated” estimates do suggest that the intervention was effective and, accordingly, future work could focus on identifying and targeting which groups would be most likely to accept the intervention to optimize its effectiveness.

Regardless, our results offer several potential lessons for quality improvement programs. First, some consideration should be given to how to provide additional support to patients with poor disease control, particularly as diet and exercise were their key challenges. Second, the timing of quality improvement interventions in reference to prior laboratory tests or upcoming office visits should be optimized. Similarly, these programs could be improved by integrating the delivery of the intervention better into clinical care, such as being delivered by pharmacists embedded within practices, particularly given the increase in risk-sharing contracts between health insurers and practices. Third, while this program did have some evening hour availability, these programs should consider exploring when would be best to outreach to patients to avoid any workplace conflicts.

There are several limitations that should be acknowledged. Our evaluation of HbA1c is limited to patients for whom Horizon had baseline laboratory availability, and laboratory values may not have been missing completely at random during follow-up. Second, the acceptance rate was on the lower range of what we estimated in our initial power calculations, possibly because the estimates were based on slightly different patient populations. Third, the adherence outcomes were measured indirectly based on administrative data, although this approach correlates well with electronic monitoring and patient self-report and is used by health plans and Medicare.[38] Medication adherence was also relatively high already in this population, which may have reduced the ability to observe an effect. Fourth, there were also slightly more men than women included on average in this trial, though this was not by design. Fifth, we were also unable to measure insulin sensitivity and C-peptide results in this study, although we would not expect these to differ between the study arms. Finally, an ideal qualitative follow-up study would have directly studied patients; however, study investigators were not to directly contact patients under the study protocol and approvals. Therefore, the detailed notes from the trained clinical pharmacists documented during their consultations were the best option to elicit factors to potentially explain the effectiveness of the intervention.

## Conclusion

A novel telephone-based patient-centered intervention did not improve HbA1c in patients with poorly-controlled diabetes compared with usual care in a large commercial insurer. The most common reason that patients identified for their lack of disease control was poor diet or exercise and that despite having poorly-controlled disease, 25% of patients were not ready to change the way they currently manage their diabetes or their regimen. These findings offer lessons for other quality improvement programs.

## Supporting information

S1 Table Pre-randomization characteristics among patients in the intervention arm by receipt of clinical pharmacist intervention. (DOCX)

S2 Table Secondary analyses of hemoglobin A1c outcomes. (DOCX)

S3 Table Patient characteristics after propensity score matching. (DOCX)

S4 Table As-treated analyses using propensity score matching. (DOCX)

S5 Table Subgroup analyses of HbA1c. (DOCX)

S6 Table Mean change in HbA1c by selected strategy in the intervention arm. (DOCX)

S7 Table Secondary analyses of medication adherence. (DOCX)

S1 Checklist CONSORT 2010 checklist of information to include when reporting a randomised trial. (DOC)

S1 Protocol Study protocol for ENGAGE-DM: ENhancing outcomes through Goal Assessment and Generating Engagement in Diabetes Mellitus. (PDF)
