# Supplementary material for: Impact of a novel pharmacist-delivered behavioral intervention for patients with poorly-controlled diabetes: The ENhancing outcomes through Goal Assessment and Generating Engagement in Diabetes Mellitus (ENGAGE-DM) pragmatic randomized trial
Source: PLoS One. 2019 Apr 2;14(4):e0214754. doi: 10.1371/journal.pone.0214754 (PMC6445420; doi:10.1371/journal.pone.0214754)
Supplement: S1 Table — (DOCX) [file pone.0214754.s001.docx]

**S1 Table. Pre-randomization characteristics among patients in the intervention arm by receipt of clinical pharmacist intervention**

| **Baseline characteristics** | **Did not receive intervention (n=476)** | **Received intervention (n=202)** |
| --- | --- | --- |
| **Demographic** |  |  |
| Age, mean (SD) | 54.8 (8.4) | 55.4 (7.5) |
| Female gender, % | 32.4 | 40.6 |
| **Diabetes values** |  |  |
| HbA1c, mean (SD) | 9.4 (1.6) | 9.2 (1.6) |
| **Oral hypoglycemic use and adherence** |  |  |
| No. oral hypoglycemics, mean (SD) | 2.1 (1.0) | 2.1 (1.0) |
| Concomitant non-insulin injectable, % |  |  |
| Adherence, mean (SD) | 81.1 (20.7) | 81.3 (18.8) |
| Copayment, mean (SD) | 39.3 (73.3) | 30.1 (63.4) |
| Type of medication |  |  |
| Generic only, % | 47.3 | 49.0 |
| Mixture, % | 41.2 | 37.1 |
| Brand only, % | 11.6 | 13.9 |
| **Diabetes characteristics, %** |  |  |
| Hypoglycemia | 0.4 | 0.5 |
| Retinopathy | 3.4 | 3.5 |
| Neuropathy | 54.0 | 55.9 |
| **Other clinical characteristics, %** |  |  |
| Coronary artery disease | 11.3 | 15.4 |
| Hypertension | 72.5 | 70.3 |
| Hyperlipidemia | 67.4 | 68.8 |
| Congestive heart failure | 0.2 | 2.5 |
| Stroke/Transient ischemic attack | 2.9 | 6.4 |
| Obesity | 27.3 | 28.2 |
| Asthma/COPD | 9.0 | 11.4 |
| Liver disease | 8.0 | 8.9 |
| Chronic kidney disease | 49.4 | 51.5 |
| Depression | 5.3 | 3.5 |
| Acute stress | 1.5 | 2.0 |
| Combined comorbidity score, mean (SD) | 0.5 (1.3) | 0.8 (1.7) |
| **Resource utilization** |  |  |
| ER visits, mean (SD) | 0.2 (0.6) | 0.3 (0.6) |
| No. of days hospitalized, mean (SD) | 0.5 (3.1) | 0.5 (3.9) |
| Office visits, mean (SD) | 6.6 (5.0) | 8.0 (5.7) |

Abbreviations: SD, Standard deviation; COPD, chronic obstructive pulmonary disease; ER, Emergency room; HbA1c, glycosylated hemoglobin A1c
