# Supplementary material for: Impact of a novel pharmacist-delivered behavioral intervention for patients with poorly-controlled diabetes: The ENhancing outcomes through Goal Assessment and Generating Engagement in Diabetes Mellitus (ENGAGE-DM) pragmatic randomized trial
Source: PLoS One. 2019 Apr 2;14(4):e0214754. doi: 10.1371/journal.pone.0214754 (PMC6445420; doi:10.1371/journal.pone.0214754)
Supplement: S2 Table — (DOCX) [file pone.0214754.s002.docx]

**S2 Table. Secondary analyses of hemoglobin A1c outcomes**

| **Outcome** | **Usual Care** | **Intervention** | **Unadjusted** | **Adjusted^*^** |
| --- | --- | --- | --- | --- |
|  |  |  |  |  |
| **Change in HbA1c: Complete case** | | | **Absolute difference (95% CI)** | |
| No. of patients  Change in HbA1c from  baseline, mean (SD)^§^ | 488  -0.79 (2.01) | 485  -0.75 (1.96) | -  +0.04 (-0.22, 0.30) | -  +0.06 (-0.20, 0.32) |
| **Proportion achieving HbA1c <8%: Complete case** | | | **Odds ratio (95% CI)** | |
| No. of patients  HbA1c <8%, % | 488  43.9% | 485  41.2% | -  0.90 (0.70, 1.16) | -  0.89 (0.69, 1.15) |
| *Adjusted for sex and prior stroke/transient ischemic attack | | | | |
| Abbreviations: HbA1c, glycosylated hemoglobin A1c; SD, Standard Deviation; CI, Confidence interval | | | | |
