# Supplementary material for: Impact of a novel pharmacist-delivered behavioral intervention for patients with poorly-controlled diabetes: The ENhancing outcomes through Goal Assessment and Generating Engagement in Diabetes Mellitus (ENGAGE-DM) pragmatic randomized trial
Source: PLoS One. 2019 Apr 2;14(4):e0214754. doi: 10.1371/journal.pone.0214754 (PMC6445420; doi:10.1371/journal.pone.0214754)
Supplement: S3 Table — (DOCX) [file pone.0214754.s003.docx]

**S3 Table. Patient characteristics after propensity score matching**

| **Baseline characteristics** | **Usual Care (n=196)** | **Intervention (n=196)** | **Absolute standardized differences** |
| --- | --- | --- | --- |
| **Demographic** |  |  |  |
| Age, mean (SD) | 55.4 (7.6) | 55.3 (7.5) | 0.01 |
| Female gender, % | 38.8 | 40.8 | 0.05 |
| **Diabetes values** |  |  |  |
| HbA1c, mean (SD) | 9.2 (1.7) | 9.2 (1.6) | 0.03 |
| **Oral hypoglycemic use and adherence** |  |  |  |
| No. oral hypoglycemics, mean (SD) | 2.1 (1.0) | 2.1 (0.9) | 0.02 |
| Concomitant non-insulin injectable, % |  |  |  |
| Adherence, mean (SD) | 80.2 (22.3) | 81.4 (18.9) | 0.03 |
| Copayment, mean (SD) | 33.6 (65.7) | 29.9 (63.2) | 0.01 |
| Type of medication |  |  |  |
| Generic only, % | 50.5 | 48.0 | 0.06 |
| Mixture, % | 36.7 | 37.8 | 0.03 |
| Brand only, % | 12.8 | 14.2 | 0.06 |
| **Diabetes characteristics, %** |  |  |  |
| Hypoglycemia | 0.5 | 0.0 | 0.10 |
| Retinopathy | 2.6 | 3.1 | 0.04 |
| Neuropathy | 55.1 | 55.1 | 0.00 |
| **Other clinical characteristics, %** |  |  |  |
| Coronary artery disease | 13.3 | 14.8 | 0.06 |
| Hypertension | 65.8 | 69.9 | 0.09 |
| Hyperlipidemia | 67.4 | 67.9 | 0.12 |
| Congestive heart failure | 2.0 | 1.5 | 0.05 |
| Stroke/Transient ischemic attack | 6.6 | 5.6 | 0.06 |
| Obesity | 31.6 | 28.1 | 0.09 |
| Asthma/COPD | 12.2 | 10.7 | 0.06 |
| Liver disease | 10.2 | 8.2 | 0.09 |
| Chronic kidney disease | 51.0 | 51.0 | 0.01 |
| Depression | 4.6 | 3.6 | 0.06 |
| Acute stress | 2.6 | 2.0 | 0.05 |
| Combined comorbidity score, mean (SD) | 0.8 (1.5) | 0.8 (1.6) | 0.01 |
| **Resource utilization** |  |  |  |
| ER visits, mean (SD) | 0.4 (1.1) | 0.3 (0.6) | 0.11 |
| No. of days hospitalized, mean (SD) | 0.4 (2.3) | 0.3 (1.8) | 0.02 |
| Office visits, mean (SD) | 8.1 (7.6) | 7.9 (5.7) | 0.04 |

Abbreviations: SD, Standard deviation; COPD, chronic obstructive pulmonary disease; ER, Emergency room; HbA1c, glycosylated hemoglobin A1c
