# Supplementary material for: Impact of a novel pharmacist-delivered behavioral intervention for patients with poorly-controlled diabetes: The ENhancing outcomes through Goal Assessment and Generating Engagement in Diabetes Mellitus (ENGAGE-DM) pragmatic randomized trial
Source: PLoS One. 2019 Apr 2;14(4):e0214754. doi: 10.1371/journal.pone.0214754 (PMC6445420; doi:10.1371/journal.pone.0214754)
Supplement: S4 Table — (DOCX) [file pone.0214754.s004.docx]

**S4 Table. As-treated analyses using propensity score matching**

| **Outcome** | **Usual Care (n=196)** | **Intervention (n=196)** | **Unadjusted** | **Adjusted^*^** |
| --- | --- | --- | --- | --- |
|  |  |  |  |  |
| ***Primary Outcome*** | | | | |
| **Change in HbA1c** | | | **Absolute difference (95% CI)** | |
| Change in HbA1c from baseline, mean (SD)^§^ | -0.48 (1.73) | -0.96 (1.69) | -0.48 (-0.91, -0.05) | -0.50 (-0.86, -0.02) |
| ***Secondary Outcomes*** | | | | |
| **Proportion achieving optimal HbA1c** | | | **Odds ratio (95% CI)** | |
| HbA1c<8.0% in follow-up^§^, % | 34.7% | 46.1% | 1.37 (0.86, 2.17) | 1.41 (0.89, 2.27) |
| **Medication adherence: PDC** |  |  | **Absolute difference (95% CI)** | |
| Adherence to ≥1 oral glucose lowering medication, mean (SD) | 80.2 (31.8) | 84.6 (27.5) | +4.4 (-1.4, 10.3) | +4.4 (-1.5, 10.3) |
| **Proportion achieving optimal adherence (PDC ≥80%)** | | | **Odds ratio (95% CI)** | |
| ≥1 medication, % | 71.4% | 76.5% | 1.30 (0.83, 2.05) | 1.29 (0.82, 2.04) |
| *Adjusted for emergency room visits (Absolute standardized difference >0.1, see Appendix Table 3) | | | | |
| ^§^Using multiple imputation (29.6% and 22.4% missing in usual care and intervention, respectively)  Note: Post-matching C-statistic: 0.590  Abbreviations: HbA1c, glycosylated hemoglobin A1c; CI, Confidence interval; SD, Standard Deviation; PDC, proportion of days covered | | | | |
