# Supplementary material for: Impact of a novel pharmacist-delivered behavioral intervention for patients with poorly-controlled diabetes: The ENhancing outcomes through Goal Assessment and Generating Engagement in Diabetes Mellitus (ENGAGE-DM) pragmatic randomized trial
Source: PLoS One. 2019 Apr 2;14(4):e0214754. doi: 10.1371/journal.pone.0214754 (PMC6445420; doi:10.1371/journal.pone.0214754)
Supplement: S5 Table — (DOCX) [file pone.0214754.s005.docx]

**S5 Table. Subgroup analyses of HbA1c**

| **Subgroup** | **Number of patients** | **Usual Care, Mean (SD)** | **Intervention, Mean (SD)** | **Relative change (95% CI)^*^** | **Interaction p-value** |
| --- | --- | --- | --- | --- | --- |
|  |  |  |  |  |  |
| Overall | 1362 | -0.79 (2.01) | -0.75 (1.96) | +0.04 (-0.22, 0.30) | N/A |
|  |  |  |  |  |  |
| Age ≥55 years | 778 | -0.70 (1.89) | -0.62 (1.83) | +0.07 (-0.25, 0.40) | 0.70 |
| Age <55 years | 584 | -0.90 (2.15) | -0.92 (2.10) | -0.01 (-0.39, 0.36) |  |
|  |  |  |  |  |  |
| Female Sex | 510 | -0.71 (2.06) | -0.39 (1.81) | +0.32 (-0.07, 0.72) | 0.09 |
| Male Sex | 852 | -0.84 (1.98) | -0.94 (2.00) | -0.10 (-0.42, 0.22) |  |
|  |  |  |  |  |  |
| Baseline HbA1c <9 | 699 | -0.06 (1.44) | -0.03 (1.47) | +0.03 (-0.26, 0.31) | 0.94 |
| Baseline HbA1c ≥9 | 663 | -1.53 (2.23) | -1.49 (2.11) | +0.04 (-0.35, 0.44) |  |
|  |  |  |  |  |  |
| Baseline adherence (PDC) <0.80 | 515 | -0.68 (2.15) | -0.75 (2.15) | -0.06 (-0.48, 0.35) | 0.52 |
| Baseline adherence (PDC) ≥0.80 | 817 | -0.84 (1.92) | -0.75 (1.82) | +0.09 (-0.22, 0.41) |  |
|  |  |  |  |  |  |
| ≥2 baseline oral hypoglycemics | 968 | -0.80 (1.96) | -0.72 (1.95) | +0.08 (-0.22, 0.38) | 0.59 |
| <2 baseline oral hypoglycemics | 394 | -0.75 (2.16) | -0.80 (1.98) | -0.06 (-0.51, 0.39) |  |
| *using multiple imputation for missing outcomes  Abbreviations: SD, Standard Deviation; CI, Confidence Interval; HbA1c, glycosylated hemoglobin A1c; PDC, proportion of days covered | | | | | |
