# Supplementary material for: Impact of a novel pharmacist-delivered behavioral intervention for patients with poorly-controlled diabetes: The ENhancing outcomes through Goal Assessment and Generating Engagement in Diabetes Mellitus (ENGAGE-DM) pragmatic randomized trial
Source: PLoS One. 2019 Apr 2;14(4):e0214754. doi: 10.1371/journal.pone.0214754 (PMC6445420; doi:10.1371/journal.pone.0214754)
Supplement: S6 Table — (DOCX) [file pone.0214754.s006.docx]

| **Shared Decision** | **No. of patients** | **Change in HbA1c, mean (SD)** |
| --- | --- | --- |
|  |  |  |
| **Change in HbA1c control from baseline** |  |  |
| Adherence improvement | 32 | -1.10 (1.56) |
| Lifestyle improvement (Diet/exercise) | 92 | -1.18 (2.23) |
| Treatment intensification | 28 | -0.38 (1.58) |
| Not Ready | 50 | -0.71 (1.54) |
| Not intervened upon | 476 | -0.56 (1.92) |

**S6 Table. Mean change in HbA1c by selected strategy in the intervention arm**

Abbreviations: HbA1c, glycated hemoglobin A1c; SD, Standard deviation

p=0.29 for difference (ANOVA)
