# Supplementary material for: Impact of a novel pharmacist-delivered behavioral intervention for patients with poorly-controlled diabetes: The ENhancing outcomes through Goal Assessment and Generating Engagement in Diabetes Mellitus (ENGAGE-DM) pragmatic randomized trial
Source: PLoS One. 2019 Apr 2;14(4):e0214754. doi: 10.1371/journal.pone.0214754 (PMC6445420; doi:10.1371/journal.pone.0214754)
Supplement: S7 Table — (DOCX) [file pone.0214754.s007.docx]

**S7 Table. Secondary analyses of medication adherence**

| **Outcome** | **Usual Care (n=684)** | **Intervention (n=678)** | **Unadjusted difference**  **(95% CI)** | **Adjusted^*^ difference**  **(95% CI)** |
| --- | --- | --- | --- | --- |
|  |  |  |  |  |
| Beginning on first fill after randomization, mean (SD) | 70.9 (30.6) | 71.5 (29.1) | +0.62 (-2.56, 3.78) | +0.48 (-2.69, 3.66) |
| Average adherence to medication, mean (SD) | 66.2 (32.7) | 67.5 (31.6) | +1.25 (-2.17, 4.66) | +1.00 (-2.42, 4.40) |
| Censoring upon insulin initiation, mean (SD) | 81.8 (31.4) | 81.7 (30.3) | -0.04 (-3.32, 3.23) | -0.16 (-3.44, 3.11) |
| *Adjusted for sex and prior stroke/transient ischemic attack | | | | |
| Abbreviations: SD, Standard Deviation; CI, Confidence interval | | | | |
