# Supplementary material for: Impact of a novel pharmacist-delivered behavioral intervention for patients with poorly-controlled diabetes: The ENhancing outcomes through Goal Assessment and Generating Engagement in Diabetes Mellitus (ENGAGE-DM) pragmatic randomized trial
Source: PLoS One. 2019 Apr 2;14(4):e0214754. doi: 10.1371/journal.pone.0214754 (PMC6445420; doi:10.1371/journal.pone.0214754)
Supplement: S1 Protocol — (PDF) [file pone.0214754.s009.pdf]

ENGAGE-DM: ENhancing outcomes through Goal Assessment and Generating Engagement in Diabetes Mellitus

SUMMARY

Among patients with poorly controlled Type 2 Diabetes Mellitus (T2DM), it is often not clear whether the problem is attributable to the healthcare provider's failure to appropriately intensify therapy, the patient's non-adherence to prescribed medications, the patient's unwillingness to accept new treatments or a combination of these factors. There are several different patient engagement techniques that could be employed in this exceptionally common situation. Two of these techniques are as follows: 1) Shared decision making (SDM), which describes the collaborative process where treatment decisions are made in a two-way exchange of information integrating both the current medical evidence and the patient's needs and preferences, and the 2) Brief Negotiated Interviewing (BNI), which incorporates an active listening model of counseling to facilitate patients' evaluation of their health risks and treatment options.

Even though SDM and BNI are complementary patient engagement techniques, no data are available on the effectiveness of combining these 2 intervention approaches – especially in the management of T2DM. In addition, few studies have used telephonic methods to deliver either of these behavioral techniques. Evaluating these techniques in tandem in a telephonic manner that is scalable, cost-effective (especially compared with in-person delivery), and innovative will provide invaluable information to healthcare providers, decision-makers and insurers to improve diabetes management.

We propose a pragmatic randomized trial of patients on least one oral hypoglycemic therapy with poorly controlled disease to test the impact of combining shared decision making and

behavioral interviewing intervention, providing both discrete decision support and ongoing motivational support to encourage medication adherence. After the trial is completed, the second phase will use predictive analytics to examine whether patients' response could have been predicted based on patient characteristics and initial receptiveness to changing health behaviors. These findings will provide valuable information about which patients will benefit from the intervention moving forward. Once disseminated, the results of this study will provide multiple benefits to stakeholders, not only about the effectiveness of these patient engagement techniques, but also about how to effectively target patients in real-world settings.

Background and Significance:

Although medications can effectively reduce high blood glucose levels in type 2 diabetes (T2DM), poor disease control is common, leading to preventable complications such as stroke, heart disease, and kidney failure. Among patients with poorly controlled T2DM, it is often not clear whether the problem is attributable to the healthcare provider's failure to appropriately intensify therapy, the patient's non-adherence to prescribed medications, the patient's unwillingness to accept new treatments or a combination of these factors. There is growing evidence supporting several different patient-targeted interventions that could be employed in this exceptionally common situation. Shared decision-making (SDM) is a patient-centered approach to improve the quality of care of patients with diabetes and other chronic conditions. While shared decision-making is often employed at a single time point in time, the management of a chronic disease, such as T2DM, frequently requires ongoing follow-up and patient engagement. By contrast, behavioral interviewing techniques, such as motivational interviewing, are typically delivered longitudinally and repeatedly, but are not necessarily

designed to help patients make decisions about how to improve their own care. Even though SDM and BNI are complementary patient engagement techniques, no data are available on the effectiveness of combining these 2 intervention approaches – especially in the management of T2DM. In addition, few studies have used telephonic methods to deliver either of these behavioral techniques. Evaluating these techniques in tandem in a telephonic manner that is scalable, cost-effective (especially compared with in-person delivery), and innovative will provide invaluable information to healthcare providers, decision-makers and insurers to improve diabetes management.

Study Objectives:

The primary aim is to examine whether a two-stage process of shared decision-making and behavioral interviewing improves glycosylated hemoglobin (HbA1c) control and medication adherence among patients who have poorly-controlled diabetes. We will also develop prediction models and examine their ability to predict response to the intervention based on baseline patient characteristics, such as sociodemographic, clinical, and medication use characteristics, as well as initial receptiveness to changing health behaviors.

Study Setting and Participants:

This study will involve Horizon BCBSNJ beneficiaries who are commercially insured (aged ≥18 years) for both medical/prescription drug benefits, have recently filled an oral medication for T2DM, have a recent HbA1c lab value ≥8%, and have provided a phone number to Horizon. Patients will be excluded if they filled insulin in the previous 6 months. Patients may be on multiple medications, including non-insulin injectables.

Study Design and Intervention Components:

This pragmatic randomized trial will include 1,400 beneficiaries of Horizon Blue Cross Blue Shield of New Jersey (BCBSNJ). We plan to primarily include participants who receive care in a number of Patient-Centered Medical Homes (PCMHs) and other population health programs that collaborate with Horizon BCBSNJ. Of these 1,400 patients, 700 will be randomized to the intervention group and 700 will be randomized to the control group.

Arm 1 (Control): Patients will not be contacted in any way.

Arm 2 (Intervention): Patients will receive a decision aid mailing and will be asked to have at least 4 telephonic discussions with pharmacists. Therapeutic choices and plan will be relayed to the patient's provider.

All patients randomized to Study Arm 2 will first be sent a decision aid that will prime them for telephonic encounters with pharmacists. This mailing will include information about the study and a pillbox and be used to enhance interventional outreach efforts. This decision aid will be developed using principles of decision aid design and be based upon other decision aids that have been previously validated.

Study Schema

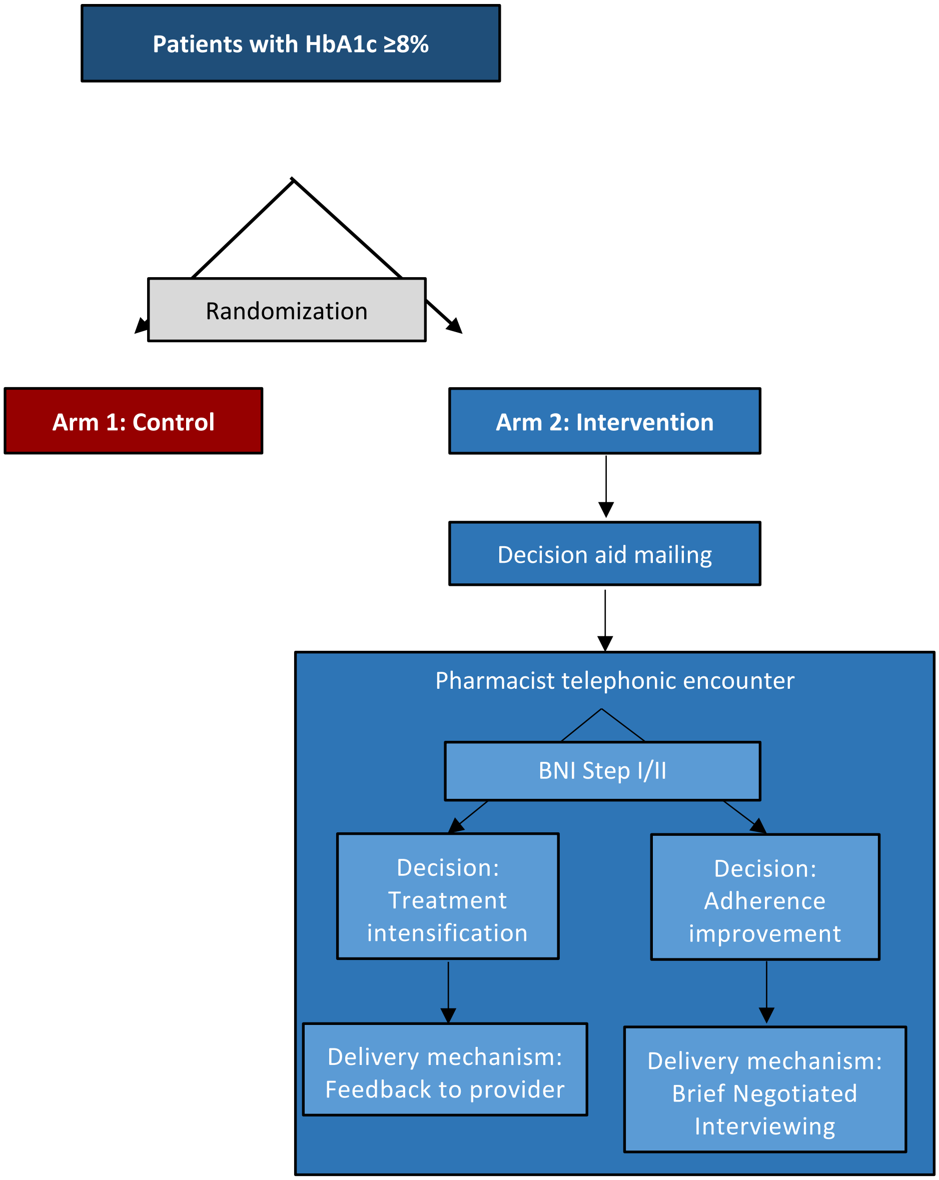

To engage patients after the initial mailing and connect them with pharmacists more quickly and directly, an Interactive Voice Response System (IVRS) may also be used. One advantage of an IVRS is that it would provide a triage format for patients to contact the pharmacists directly after receiving the initial mailing. IVRS is frequently used in clinical practices and Horizon BCBSNJ to help manage their patients.

After the initial mailings, the pharmacists will attempt to reach each patient in the intervention group at least 4 times for the initial conversation. Once a patient is reached, the Magellan pharmacist will explain the purpose of the consultation and ask the patient if he/she would like to participate (please see attached example call scripts). If a patient agrees, this will be considered implicit consent. If a patient wishes to not participate and wishes to not receive further contact, this will be noted and the patient will not be contacted again.

The first telephonic encounter with the clinical pharmacist will consist of a 2-stage process of identifying patients' motivations and driving a consensus of decision choices. For these encounters, the Magellan pharmacists will use a semi-structured call guide developed by the study team for both the initial intervention and follow-up 'booster' phone calls. These telephonic encounters will include discussions about diabetes treatment options, goals and preferences, medication adherence, strategies for reducing barriers to adherence, implementing lifestyle modifications and the benefits of maintaining blood glucose control. In these consultations, both discrete decision support and ongoing motivational support will be provided to encourage medication adherence.

In brief, the telephonic discussions with pharmacists will follow a semi-structured call guide that flows through the following phases (as part of the 2-stage process):

(a) confirm treatment regimens,

(b) discuss treatment goals and preferences,

(c) engage the patient in sharing potential medication non-adherence issues or lifestyle factors that may be contributing to poor control,

(d) discuss potential barriers and willingness/readiness to modify behaviors, and

(e) engage the patient in identifying and agreeing upon a possible shared plan of behavioral strategies to improve glucose control and potential treatment modifications.

The two stages of the call guide are summarized, as follows:

- Stage 1 Shared-Decision Making: The first stage of the intervention encounter consists of the shared decision-making process whereby discussions of issues and barriers to glucose control will be identified and discussed.
    - These discussions will occur in an open-ended manner, which will allow the patients to elaborate and problem solve as well as illuminate underlying beliefs and concerns that may affect glucose control.
    - The previously-mailed decision aid developed for telephonic use will be employed to aid in this encounter, which will to help the patient understand and reconcile the relative personal risks and benefits of medication adherence and treatment intensification to improve their disease control.

    - Ultimately, the goal of this first stage of the shared-decision engagement making process is patient involvement in the conversation and their care. The shared decisions may involve intensification of therapies for patients who are already adherent to their current regimen (as determined by patient self-report) or changing their medication adherence behaviors.

- Stage 2: Once coming to a shared decision between the pharmacist and patient about how to improve the patient's diabetes control, the second stage may involve a behavioral interviewing engagement technique if the shared decision involves adherence improvement as a goal.

    - This model incorporates an active listening model of counseling, identifying patients' readiness for change and level of behavior change using the Brief Negotiated Interview (BNI). The BNI employs some features of motivational interviewing but through a short structured interview that incorporates brief feedback and advice with motivational enhancement techniques.
    - The BNI proceeds through the following four main steps: (1) raising support; (2) providing feedback; (3) enhancing motivation through assessing readiness and developing discrepancy between behavior and goals; (4) negotiating and advising.
    - To develop the structured BNI tool within the call guide for this intervention, there are established algorithms that will be used. The goal of this stage is to motivate patients to change behaviors.

At the end of each conversation with the clinical pharmacist, a shared treatment plan will be identified, which will be modified upon each of the three subsequent encounters between the pharmacist and the patient. The barriers that will be addressed within the shared plan may include medication non-adherence but potentially also lifestyle modifications or issues with treatments, such as weight changes, low blood sugar, other side effect considerations, daily routines, any daily monitoring, and cost barriers.

The barriers and proposed plan for each patient will be communicated from the pharmacist to the patient's provider, either via letters, faxes, and phone calls, depending on urgency. Ultimately any therapeutic decision (e.g. to change or intensify treatment) will be performed by the patient's own treating physician. For patients who decide through the course of this intervention to adhere to their currently prescribed treatment, they are following treatment recommendations already set forth by their providers.

These pharmacist-delivered phone calls will occur a minimum of 4 times during the follow-up period. The follow-up "booster" phone calls will repeat some of these themes and continue to engage the patient in discussions surrounding these topics.

To enhance the secondary aim of the study, the pharmacists will capture some brief additional baseline information on patients assigned to the intervention arm during the initial call. Among patients randomized to the intervention arm, patients will be asked to respond to a few questions. These additional items are anticipated to include a set of items on self-reported

medication adherence, such as the 1-item Morisky adherence question and readiness to change. Administered towards the beginning of the initial call, these questions will be built into the semi-structured call guide used by the pharmacists. The answers to these questions will not only help provide the pharmacists with additional information that is relevant to the two patient engagement techniques (and could be captured within these conversations in a less structured or validated manner), but will also be tested in the secondary predictive modelling study to identify the types of patients who will benefit from this intervention. The answers to these questions will not be used to screen or enroll patients.

Timing of study interventions

Study Arm	Initial mailing	Initial call	Follow-up call (#1)	Follow-up call (#2)	Follow-up call (#3)
**Intervention group**	Decision aid + Educational mailing	Introduction + SDM + BNI	Booster: SDM + BNI	Booster: SDM + BNI	Booster: SDM + BNI

Outcomes:

The primary outcome of interest will be the pre- to post-intervention change in mean HbA1c levels in each treatment arm from randomization to the end of follow-up. Horizon BCBSNJ receives laboratory information from over 200 patient-centered medical homes and other population health programs. This laboratory information will be used to measure the change in HbA1c levels. We will use generalized estimating equations to compare the changes between the two study groups.

The data that will be used to analyze the impact of the study is that which will have been generated as a result of routine care, including medication prescription data, relevant laboratory results, information from the clinical pharmacist calls, and health care utilization. In specific, the HbA1c result recorded closest to the 12-month end of follow-up as provided in the laboratory data will be used for the primary analysis. For subjects with missing outcome data, multiple imputation will be used to impute missing follow-up values to calculate the change in HbA1c levels.

Secondary outcomes will include both glycemic outcomes and medication adherence outcomes. The secondary glycemic outcomes will include mean HbA1c levels and the proportion of patients achieving optimal glycemic control, defined as the proportion of patients who achieved a HbA1c <8.0%. Patients' adherence to their diabetes medications will be measured by pharmacy claims and their filling patterns. Adherence will be assessed using the proportion of days covered (PDC), or the proportion of days that patients had medication available to them during follow-up. We will also measure and examine other adherence and persistence measures

as secondary outcomes, including mean PDC in each study arm, the proportion of patients achieving optimal adherence (defined by ≥80% PDC), and gaps in medication availability.

Randomization and Sample size:

Randomization will occur in a 1:1 ratio conducted on the patient level. This randomization will occur using a random number generator at Horizon Analytics after the initial application of inclusion/exclusion criteria. The randomization key will be maintained at Horizon Analytics as well as the allocation of patients to the intervention and control arms.

We anticipate that an enrollment of at least 682 individuals in each treatment arm should be sufficient to detect an average change of 0.5% in A1c, assuming an $\alpha=0.05$, $1-\beta=0.80$, A1c standard deviation=1.9, pharmacist reach rate, and 25% loss-to-follow-up, including clustering. With this sample size, we should also have the ability to detect differences in the adherence outcomes. These are estimates based on previous literature as well as feasibility estimates from Horizon and clinical experience using an intention-to-treat perspective.

Statistical methods:

We will measure key baseline characteristics in the 12-months prior to randomization in the pharmacy and medical claims data and enrolment files. These characteristics will include sociodemographic characteristics, such as age and sex, which will be measured in the enrollment files. We will provide absolute standardized differences between the two treatment groups.

Primary outcome (change in HbA1c): In the primary analysis, the primary outcome, mean change in glycemic control, will be compared using generalized estimating equations with an identity link function (as a continuous variable) and normally distributed errors within the imputed full analysis set, as described above. Data will be presented as the unadjusted absolute difference in the change in HbA1c between the groups along with 95% confidence intervals.

Secondary outcome (proportion achieving glycemic control)

1) *Proportion of patients achieving optimal glycemic control* (defined by HbA1c<8): This outcome will be compared using generalized estimating equations with a logit link and binary distributed errors. Data will be presented as the unadjusted odds ratio for optimal glycemic control between the groups along with 95% confidence intervals.

Secondary outcomes (medication adherence):

1) M*ean PDC in each study arm:* This outcome will be compared using generalized estimating equations with an identity link function and normally distributed errors. Data will be presented as the unadjusted absolute difference between the groups along with 95% confidence intervals.

2) *Proportion of patients achieving optimal adherence* (defined by ≥0.80 PDC): This outcome will be compared using generalized estimating equations with a logit link and binary distributed errors. Data will be presented as the unadjusted odds ratio for optimal adherence between the groups along with 95% confidence intervals.

After the completion of the trial, we will use predictive analytics to examine whether the outcomes could have been predicted based on patient factors, such as sociodemographic, clinical, medication use and adherence, other self-reported motivational characteristics, and receipt of the pharmacist-delivered telephonic intervention. These predictive ability of these models will be assessed using model discrimination and performance measures, using logistic regression, boosted regression and machine learning approaches.
